# Disulfide bond status links the open and closed conformations of prothrombin

**DOI:** 10.1002/pro.70842

**Published:** 2026-11-05

**Authors:** Mark Schreuder, Diego Butera, Suresh Kumar, Nicola Pozzi, Philip J. Hogg

**Affiliations:** ^1^ School of Life Sciences University of Technology Sydney Sydney New South Wales Australia; ^2^ The Centenary Institute The University of Sydney Sydney New South Wales Australia; ^3^ Department of Biochemistry & Molecular Biology Saint Louis University School of Medicine St. Louis Missouri USA

**Keywords:** antiphospholipid syndrome, disulfide bond, protein conformation, prothrombin

## Abstract

Emerging evidence indicates that structural changes in proteins can influence autoimmune recognition. Antibodies targeting the central blood coagulation protein, prothrombin, are frequently detected in patients with the autoimmune disorder, antiphospholipid syndrome. Prothrombin exists as a dynamic equilibrium between closed and open conformations, and the open conformation is more antigenic. Using differential cysteine alkylation and mass spectrometry, we demonstrate that prothrombin circulates as an array of partially disulfide‐bonded protein states. Immunoprecipitation of prothrombin from pooled plasma with an antibody that recognizes the open conformation resulted in enrichment of prothrombin molecules containing unformed disulfide bonds. Genetic ablation of disulfides revealed that individual bonds modulate the equilibrium between prothrombin's open and closed conformations. Unformed disulfide bonds in the kringle‐1 (C65–C143 and C114–C138), kringle‐2 (C191–C231), and protease domain (C293–C439, C493–C507, and C521–C551) were associated with a shift in the protein's structure toward a more open conformation, as assessed by enhanced chymotrypsin accessibility and increased binding to the open conformation antibody. These findings indicate that the disulfide bond status of prothrombin contributes to the open and closed conformational equilibrium.

## 1 INTRODUCTION

International laboratory criteria for antiphospholipid syndrome (APS) diagnosis focus on lupus anticoagulant (LA), anti‐cardiolipin, and anti‐β2‐glycoprotein I (β2GPI), although anti‐prothrombin antibody testing is increasingly used as an additional diagnostic tool to improve risk stratification (Sciascia et al., 2019). Anti‐prothrombin antibodies, particularly antibodies against the prothrombin‐phosphatidylserine complex, are strongly associated with key clinical features of APS, including vascular thrombosis and obstetric complications (Ding et al., 2023; Sciascia et al., 2012). Several pathogenic mechanisms have been proposed to account for their prothrombotic activity (Ding et al., 2023), including a decreased inactivation due to activated protein C resistance (Kaneshige et al., 2019; Noordermeer et al., 2023; Pontara et al., 2021), an increase in tissue factor expression (Kaneshige et al., 2019; Muller‐Calleja et al., 2024; Vega‐Ostertag et al., 2006), an increase in the affinity of prothrombin for membrane binding (Rao et al., 1996), and activation of platelets through interaction with the FcγRIIA receptor (Chayoua et al., 2021). Despite their clinical relevance and recent advances in the field (de Laat et al., 2024; Pengo & Pozzi, 2025), the structural basis of prothrombin recognition by pathological autoantibodies remains incompletely understood.

Emerging evidence indicates that conformational plasticity in prothrombin contributes to autoantibody recognition in APS (Pengo & Pozzi, 2025). In circulation, prothrombin exists in a dynamic equilibrium between open and closed conformations, with approximately 80% of molecules adopting a closed conformation (Pozzi, Bystranowska, et al., 2016). The closed form is compact, characterized by stable interactions between the kringle‐1 and the serine protease domain (Chinnaraj et al., 2018). Conversely, the open conformation adopts an elongated structure in which the domains align vertically along the main axis (Pozzi, Chen, & Di Cera, 2016). These structural differences alter epitope accessibility, and a subset of anti‐prothrombin antibodies, referred to as type‐I, selectively recognize the open conformation (Chinnaraj et al., 2019).

Our recent studies have shown that proteins exist not only in different conformational states, but also in a heterogeneous mixture of disulfide states characterized by natural variation in disulfide bond formation. Disulfide bonds, covalent links between two cysteine residues, are critical for defining a protein's three‐dimensional structure, yet we have identified that several major plasma proteins, including fibrinogen (Butera & Hogg, 2020), α2‐macroglobulin (Butera & Hogg, 2020), and von Willebrand factor (Butera et al., 2023; Kutzki et al., 2023), as well as the membrane receptors αIIbβ3 (Pijning et al., 2021) and Mac‐1 (Dupuy et al., 2023), are constitutively produced as partially disulfide‐bonded forms. Similarly, others observed partially unformed disulfide bonds in factor XII (Yang et al., 2026) and IgG molecules (Gevondyan et al., 2006; Liu & May, 2012; Robotham & Kelly, 2019). In these proteins, a defined fraction of bonds remains unformed during synthesis, generating a population‐level cysteine redox heterogeneity with distinct structural and potentially functional properties.

The present study reports that prothrombin circulates in blood as an array of disulfide‐bonded states. Our studies indicate that prothrombin's disulfide bonds influence the equilibrium between the open and closed conformations, which could potentially underlie prothrombin's antigenicity in APS.

## 2 RESULTS

### 2.1 Prothrombin circulates in plasma as an array of disulfide‐bonded states

Prothrombin contains 12 disulfide bonds (Figure 1a), all of which have been structurally defined in X‐ray structures (Chinnaraj et al., 2018; Pozzi, Chen, & Di Cera, 2016). To determine the disulfide‐bonded state of circulating prothrombin, we immunoprecipitated prothrombin from healthy donor plasma and performed differential cysteine alkylation using isotopic iodophenylacetamide agents (^12^C‐IPA and ^13^C‐IPA) and mass spectrometry. The relative abundance of cysteine‐containing peptides labeled with ^12^C‐IPA and/or ^13^C‐IPA was used to quantify the proportion of each cysteine present in the unformed versus formed disulfide state (Figure S1, Table S1). Values are reported as the fraction of each cysteine that is unformed within the total circulating prothrombin population. Using this approach, we could quantify the redox state of 21 of the 24 cysteines in prothrombin, representing 11 of its 12 disulfide bonds (Figure 1b). The state of the C17–C22 disulfide in the Gla domain could not be determined.

**FIGURE 1:**
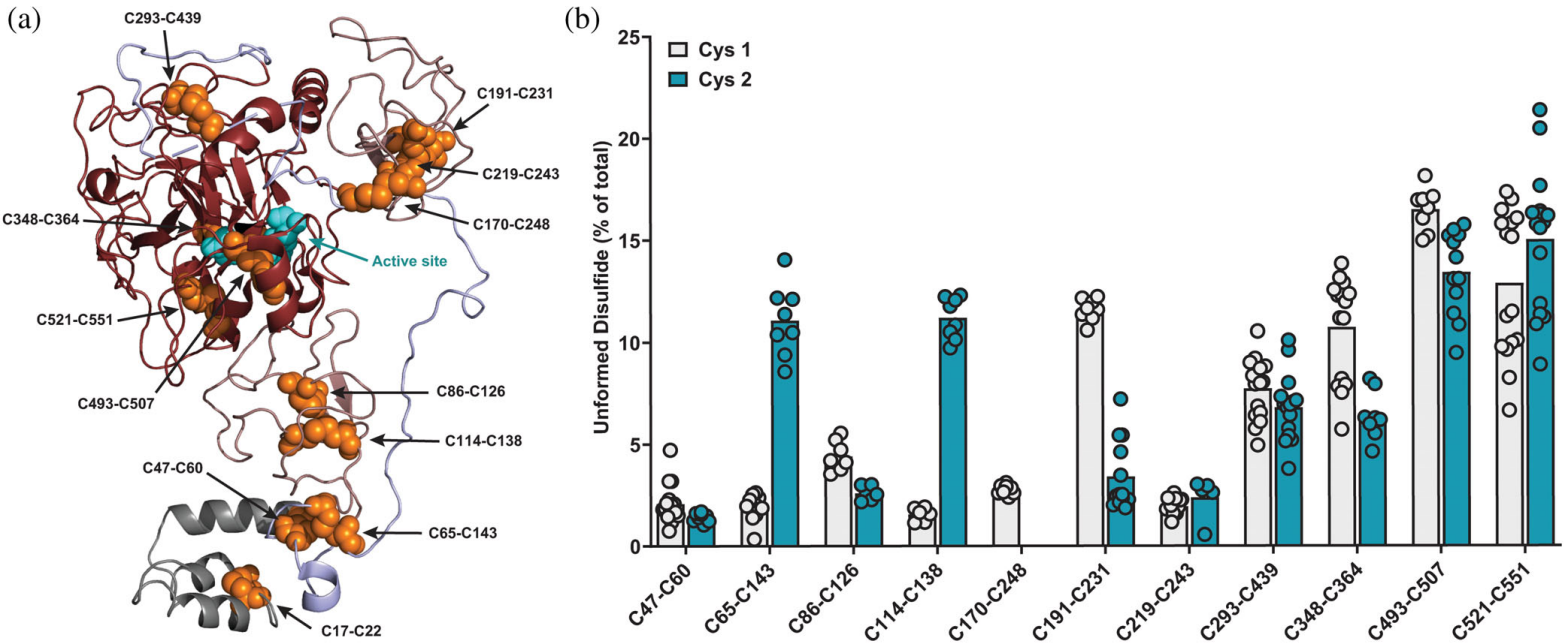
Prothrombin circulates in plasma as an array of disulfide‐bonded states. (a) The modeled structure of prothrombin (PDB ID 6C2W; Chinnaraj et al., 2018) highlights the positions of the active site and its 12 disulfide bonds. Orange spheres represent the disulfide bonds, with arrows indicating the corresponding cysteine residue numbers. The active site, comprising His363, Asp419, and Ser525, is shown as cyan spheres. The Gla domain is shown in gray, the linkers in light blue, the kringle‐1 and kringle‐2 domains in dark salmon, and the protease domain in chocolate. (b) The states of 11 disulfide bonds in prothrombin were analyzed from plasma samples of eight healthy human donors. For each disulfide bond, the two constituent cysteines are shown separately in numerical order (Cys 1 and Cys 2), reflecting independent quantification of each cysteine‐containing peptide by mass spectrometry. The redox state of the cysteines forming the C17‐C22 disulfide bond could not be determined. Round symbols represent the redox state of individual cysteines from each donor, while bars indicate the mean redox state across all donors.

While several disulfide bonds were predominantly formed (<5% unformed), all bonds existed as mixtures of formed and unformed states. Notably, multiple disulfides, particularly those within the serine protease domain, were unformed in a substantial fraction of circulating molecules, ranging from 1 in 10 to 1 in 5 of the total population. These findings establish that prothrombin circulates in plasma as a heterogeneous mixture of disulfide‐bonded states, indicating the presence of structural variability within the prothrombin population. The coexistence of formed and unformed disulfide bonds across the molecule, together with the combinatorial possibilities, suggests the potential for numerous distinct covalent forms. However, because our analysis reports population‐level redox states of individual cysteines, the specific combinations of disulfide bonds within single molecules cannot be directly resolved.

Interestingly, the two cysteines of the disulfide bonds C65–C143, C114–C138, and C191–C231 displayed markedly different redox states. This could indicate the presence of alternative covalent configurations in a subset of molecules. For example, based on our data, it is theoretically possible that C65 and C114 form a non‐canonical disulfide bond, which would account for their more oxidized state relative to their conventional partners C143 and C138, respectively. However, disulfide‐linked peptide analysis did not detect a substantial population of alternative disulfide pairings, although such species are difficult to detect and quantify by mass spectrometry. An alternative explanation is that free cysteines undergo post‐translational modifications that block ^12^C‐IPA labeling. During the analytical workflow, reduction with DTT would remove these modifications (Casadei et al., 2008), enabling subsequent labeling with ^13^C‐IPA. In such cases, modified free cysteines would appear artificially enriched in the oxidized state.

### 2.2 An open prothrombin conformation antibody selectively binds specific disulfide‐bonded subsets

Prothrombin circulates in an equilibrium between closed and open conformations, but the role of the disulfide bonds remains unknown. The monoclonal antibody, POmAb, recognizes a kringle‐1 epitope exposed only in the open conformation of prothrombin (Kumar et al., 2024). We used POmAb to immunoprecipitate prothrombin from normal pooled plasma and compared the disulfide‐bond states of the bound and unbound molecules.

The POmAb‐bound fraction was enriched in prothrombin molecules containing unformed disulfide bonds relative to both the total plasma prothrombin pool and the unbound fraction (Figure 2). These findings indicate that POmAb preferentially recognizes a subset of prothrombin molecules defined by their disulfide‐bonded state, demonstrating that not all covalent states are structurally equivalent with respect to antibody binding. These data therefore reflect selective enrichment of pre‐existing conformational states rather than antibody‐induced shifts in the open–closed equilibrium.

**FIGURE 2:**
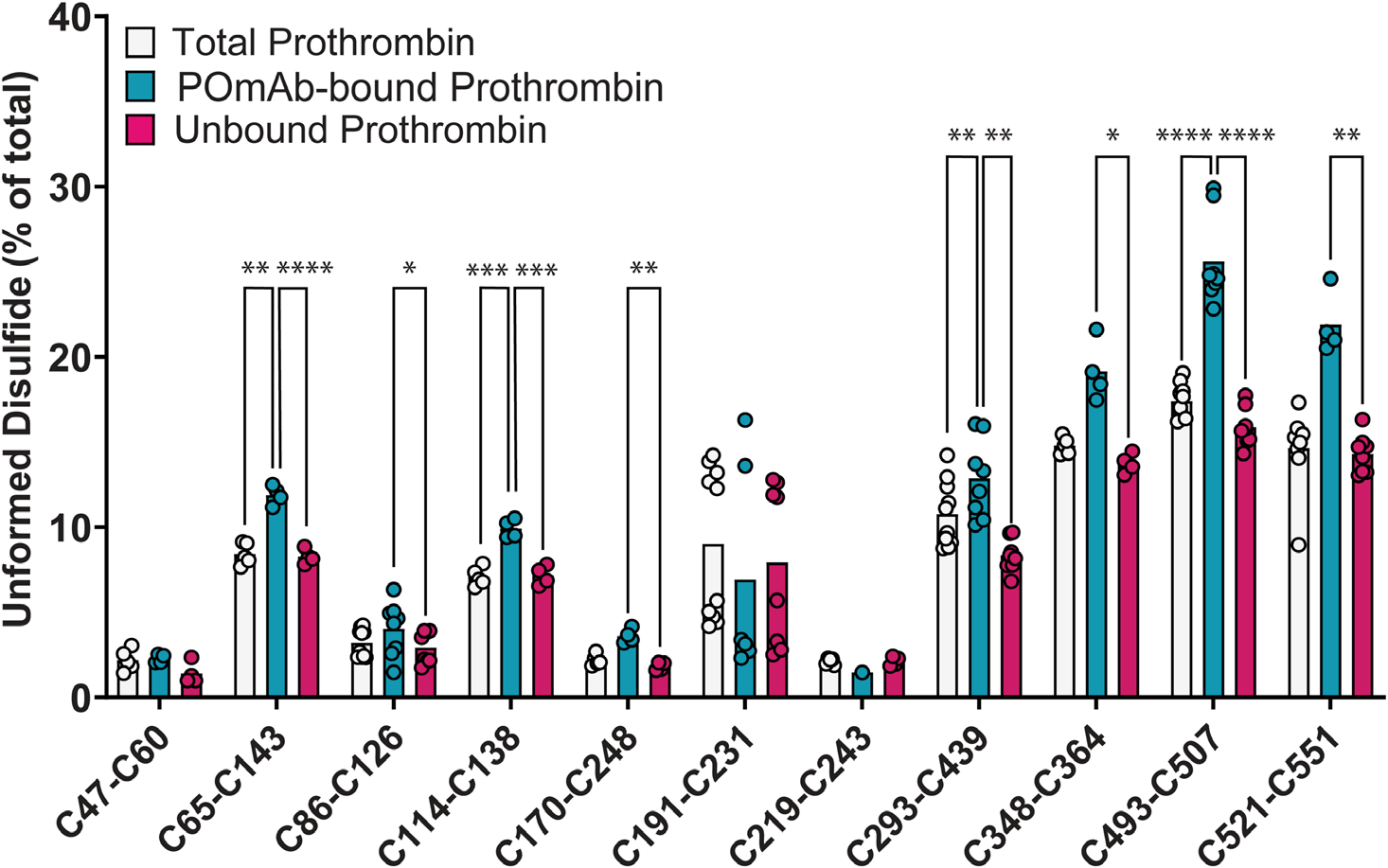
An open prothrombin conformation antibody preferentially recognizes specific disulfide‐bonded subsets. Disulfide bond state of prothrombin from normal pooled plasma immunoprecipitated by POmAb. Shown are samples taken from plasma representing the total prothrombin pool, POmAb‐bound prothrombin, and the prothrombin fraction that remained unbound during immunoprecipitation. Each dot represents the state of an individual cysteine from one sample, while bars indicate the mean redox state across all samples. *p < 0.05; **p < 0.01; ***p < 0.001; ****p < 0.0001 according to multiple paired *t*‐tests analysis.

### 2.3 Ablation of prothrombin disulfide bonds preserves procoagulant activity

To investigate the structural and functional relevance of prothrombin's heterogeneous mix of covalent states, we generated a panel of variants (Figure 3a) in which the two cysteines forming the most frequently unformed disulfide bonds were substituted with alanine to abolish disulfide bond formation while minimizing the introduction of new side‐chain chemistry or hydrogen‐bonding interactions. The prothrombin variants were stably expressed in HEK293T cells and purified to homogeneity by ion‐exchange chromatography as described in the Materials and Methods section. The method of purification of the recombinant prothrombin's separated un‐ or under‐carboxylated proteins from fully carboxylated molecules (Camire et al., 2000; Larson et al., 1998).

**FIGURE 3:**
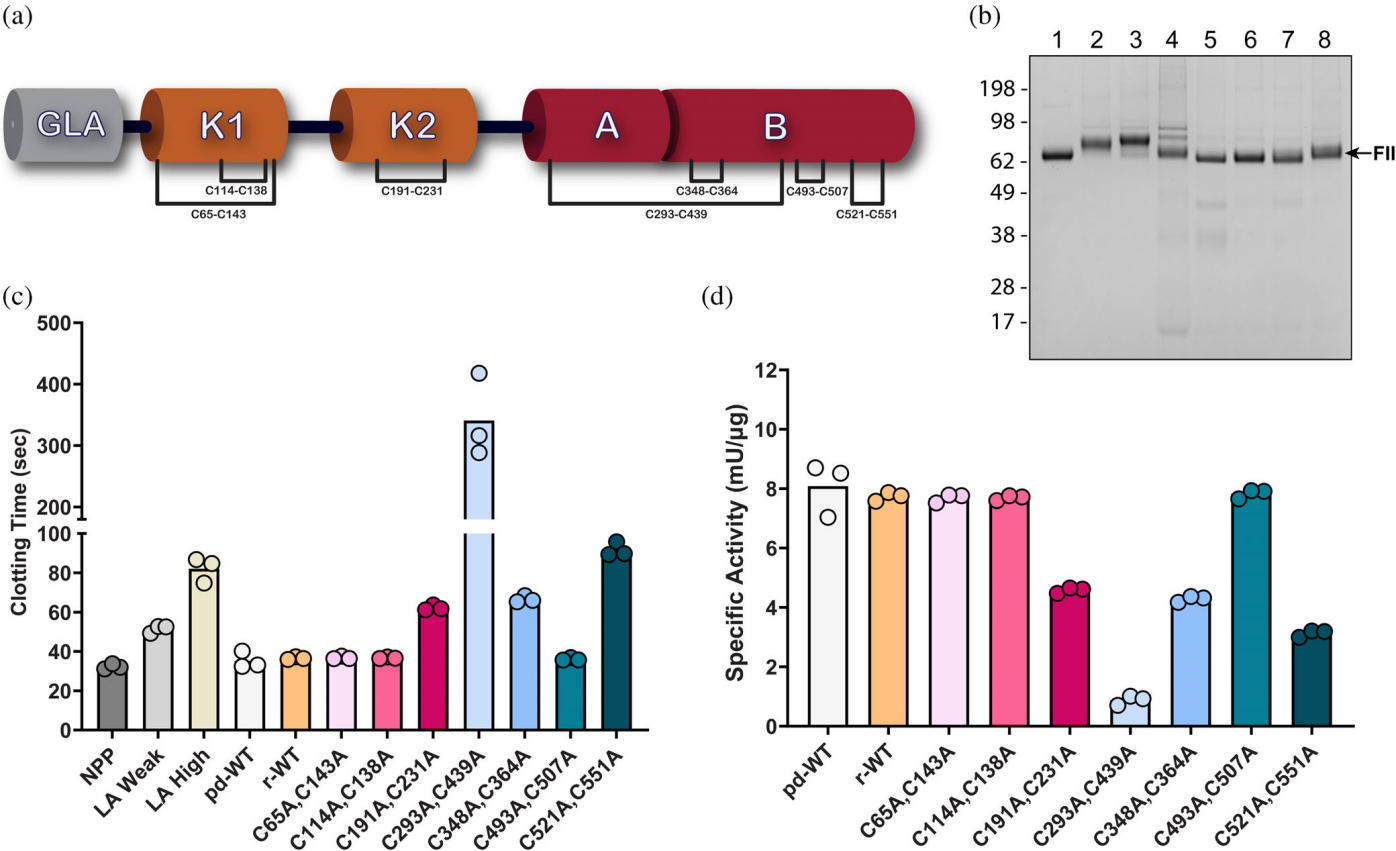
Ablation of prothrombin disulfide bonds preserves procoagulant activity. (a) A schematic representation of prothrombin composed of the Gla domain (GLA), the kringle‐1 and 2 domains (K1 and K2, respectively), and a serine protease domain containing the A and B chains. Black lines connecting the domains represent the linker regions. The disulfide bonds that were mutated in this study through double cysteine‐to‐alanine mutation are indicated. (b) SDS‐PAGE of purified prothrombin variants (1 μg per lane) under non‐reducing conditions visualized by staining with Coomassie Brilliant Blue R‐250. Lane 1, wild‐type (WT); lane 2, C65A,C143A; lane 3, C114A,C138A; lane 4, C191A,C231A; lane 5, C293A,C439A; lane 6, C348A,C364A; lane 7, C493A,C507A; lane 8, C521A,C551A. Relevant prothrombin (FII) bands and the apparent molecular weights of the standards are indicated. (c,d) The clotting times (c) and specific activities (d) of indicated prothrombin variants were assessed by a lupus anticoagulant‐sensitive activated partial thromboplastin time‐based assay. Specific activities were calculated using a serial diluted reference curve from normal control plasma, assuming 100 μg/mL prothrombin equals 1 Unit. NPP refers to normal control plasma, LA Weak and High refer to control plasmas with low or high lupus anticoagulant activity, respectively. pd‐WT refers to plasma‐derived wild‐type prothrombin, r‐WT refers to recombinant wild‐type prothrombin. Each dot represents a single measurement, while bars indicate the mean across samples. *p < 0.05; ****p < 0.0001 compared to pd‐WT according to one‐way ANOVA and Dunnett's multiple comparison test.

Examination of the prothrombin mutant disulfide states revealed that ablation of one disulfide bond did not significantly alter the state of other disulfide bonds, indicating that disulfide formation in prothrombin is not interdependent (Figure S2). The electrophoretic mobility of each variant was analyzed by SDS–PAGE under non‐reducing (Figure 3b) and reducing (Figure S3) conditions. Under non‐reducing conditions, variants lacking the kringle‐1 disulfide bond C65–C143 or C114–C138 migrated slower than wild‐type prothrombin, suggesting that when denatured, differences in the disulfide‐bond state may induce different conformations. Unexpectedly, this aberrant migration persisted under reducing conditions, indicating that the mobility shift was not attributable to differences in disulfide bonding. To determine whether post‐translational glycosylation differences accounted for the slower migration, the variants were treated with Peptide‐N‐Glycosidase F. However, the slower migration remained after enzymatic removal of N‐linked glycans, excluding differential N‐linked glycosylation as the underlying cause (Figure S4). Although potential O‐linked glycosylation differences cannot be fully excluded, prothrombin does not comprise O‐linked glycans, and cysteine‐to‐alanine mutations are not known to introduce new glycosylation sites. Therefore, these findings suggest that mutation of the kringle‐1 disulfide bonds results in physicochemical differences that are retained even under denaturing and reducing conditions.

To determine whether disulfide bond mutations disrupted procoagulant function, the variants were tested in a LA‐sensitive activated partial thromboplastin time (aPTT) assay. Prothrombin‐deficient plasma was supplemented with each recombinant variant at 100 μg/mL, and clotting times were compared with those obtained using purified plasma‐derived prothrombin, normal control plasma, and LA control plasmas (Figure 3c). Recombinant wild‐type prothrombin produced clotting times comparable to purified plasma‐derived prothrombin, indicating that the recombinant protein retained normal procoagulant function. Variants lacking the C65–C143, C114–C138, or C493–C507 disulfide bonds showed similar clotting times to wild‐type, suggesting that ablation of these bonds does not substantially impair clotting activity. In contrast, the C191A,C231A, C348A,C364A, and C521A,C551A variants exhibited twofold to threefold prolonged clotting times, consistent with reduced efficiency of prothrombin activation and/or diminished thrombin activity. The C293A,C439A variant showed a markedly prolonged clotting time (approximately 10‐fold), indicating that ablation of this disulfide bond severely compromises prothrombin function. To facilitate comparison between variants, clotting times were converted to specific activity values using a standard curve generated from serial dilutions of normal control plasma (Figure 3d). This analysis showed that most disulfide bond variants retained substantial procoagulant activity, whereas the C293A,C439A variant displayed severely reduced activity.

### 2.4 Prothrombin disulfide bond status modulates the equilibrium between the open and closed conformations

The contribution of disulfide bond state to the closed/open conformational transition in prothrombin was examined using two different assays: limited chymotrypsin proteolysis and kinetics of binding of the open conformation POmAb.

In the closed conformation, a stabilizing interaction between kringle‐1 residue Tyr93 and Trp547 in the protease domain shields the chymotrypsin cleavage site at Trp468 (Chinnaraj et al., 2018; Pozzi, Bystranowska, et al., 2016). This site becomes accessible in the open form. Following incubation of prothrombin variants with chymotrypsin, reactions were applied to SDS‐PAGE and protein fragments were visualized by Coomassie blue staining (Figure 4a). Full‐length prothrombin bands were analyzed by densitometry and depicted as the percentage of full‐length prothrombin following chymotrypsin treatment relative to prothrombin samples in the absence of chymotrypsin (Figure 4b). As expected, prothrombin wild‐type underwent minimal proteolysis due to its predominantly closed conformation (Figure 4a,b). Similarly, chymotrypsin proteolysis was largely absent for prothrombin C348A,C364A. Strikingly, six of the seven disulfide bond mutants displayed robust proteolysis by chymotrypsin, consistent with a shift toward the open conformation. It is important to note that disulfide bond mutation could have altered prothrombin's structure and exposed an otherwise hidden chymotrypsin cleavage site that is unrelated to the open and closed state.

**FIGURE 4:**
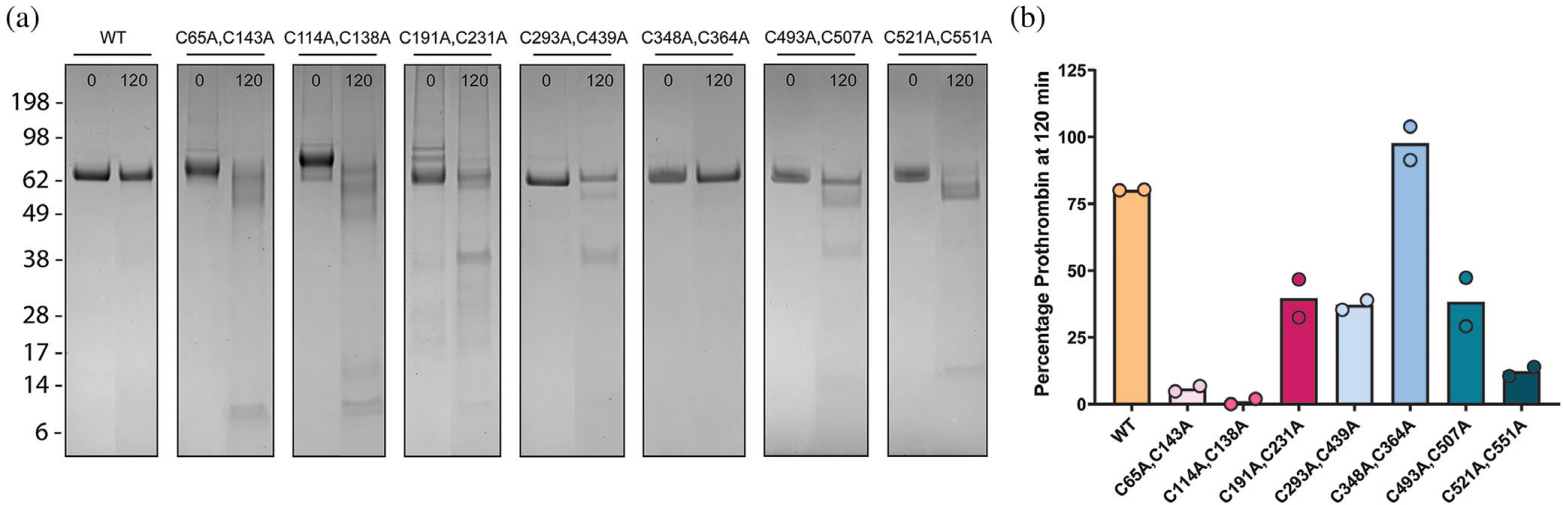
Prothrombin disulfide bond status modulates the equilibrium between open and closed conformations. (a) SDS‐PAGE of purified prothrombin variants (0.1 mg/mL) subjected to limited proteolysis by chymotrypsin (1:180 (w/w)) quenched at 0 and 120 min. (b) Densitometry analysis of the full‐length prothrombin bands shown by SDS‐PAGE in (a) indicating the percentage of non‐proteolyzed prothrombin remaining following chymotrypsin treatment at 120 min.

To avoid potential confounding effects of disulfide bond mutation‐induced chymotrypsin cleavage sites, we used surface plasmon resonance binding to POmAb as a protease‐independent assessment of the closed–open equilibrium (Kumar et al., 2024). As expected, wild‐type prothrombin bound POmAb only weakly, reflecting its ~80% closed conformation (Figure 5a). In accordance with the minimal chymotrypsin proteolysis, the C348A,C364A mutant gave essentially identical POmAb binding compared to wild‐type (Figure 5a,b; Table 1), indicating that this protease‐domain bond does not appreciably alter the equilibrium. By contrast, mutants lacking C191–C231, C293–C439, C493–C507, or C521–C551 showed a threefold to ninefold increased POmAb binding affinity (Figure 5a,b, Table 1). This increased binding affinity was the result of an increased prothrombin–POmAb association rate (Table 1). As such, this surface plasmon resonance data provides further evidence that selective disulfide modulation can alter the closed–open conformational equilibrium.

**FIGURE 5:**
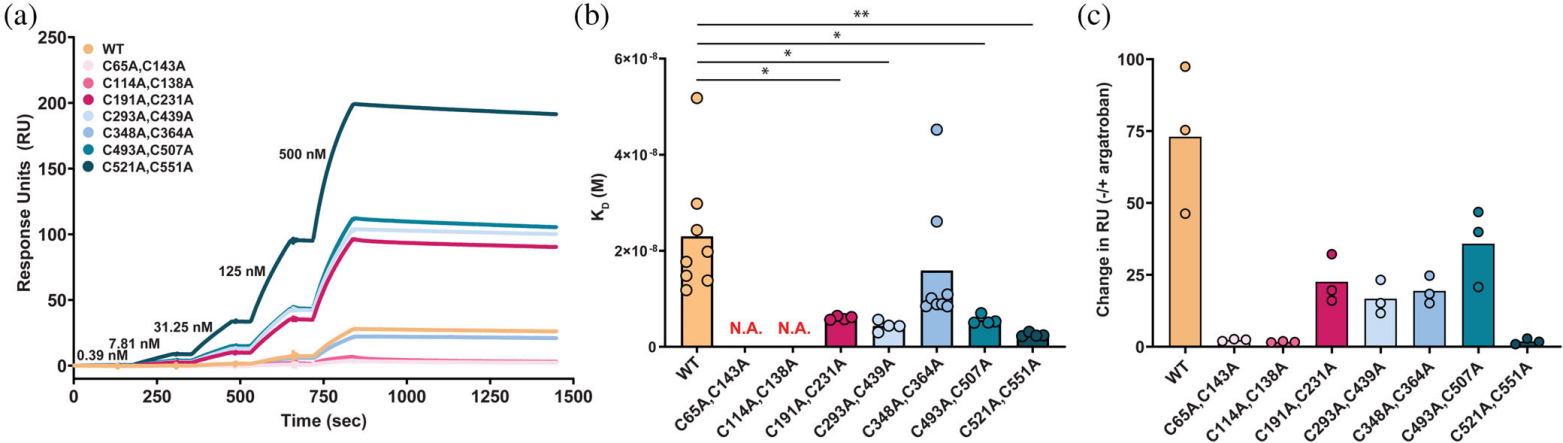
Prothrombin disulfide bond status modulates the binding to an open conformation monoclonal antibody. (a) Baseline‐subtracted association and dissociation sensorgrams displaying prothrombin binding to the prothrombin open conformation monoclonal antibody (POmAb). Increasing concentrations (0.39–500 nM) of indicated prothrombin variants were injected onto a CM5 chip with immobilized POmAb. The data are representative of four to eight independent experiments. (b) Binding affinities of prothrombin variants for POmAb calculated from the on and off rates obtained from the sensorgrams obtained in (a). Statistical significance: *p < 0.05; **p < 0.01 according to one‐way ANOVA analysis. N.A., for this experiment, binding was too low to accurately assess kinetic parameters. (c) Increase in response units (RU) for prothrombin variants binding to POmAb in the presence of the active site ligand argatroban relative to conditions in the absence of argatroban. The data are representative of three independent experiments.

**TABLE 1:** Binding kinetics of prothrombin variants to the open conformation antibody POmAb or the active site ligand argatroban measured by surface plasmon resonance.

Prothrombin	Prothrombin·POmAb			Prothrombin·argatroban		
	*k*_on_ (M^−1^ s^−1^)	*k*_off_ (s^−1^)	*K*_D_ (M)	*k*_on_ (M^−1^ s^−1^)	*k*_off_ (s^−1^)	*K*_D_ (M)
WT	7.07E+03 ± 2.69E+03	1.26E‐04 ± 1.57E‐05	2.20E‐08 ± 1.38E‐08	4.72E+04	5.24E‐01	3.04E‐05
C65A,C143A	N.A.	N.A.	N.A.	1.27E+05	2.70E‐01	3.96E‐06
C114A,C138A	N.A.	N.A.	N.A.	3.08E+05	7.05E‐01	4.27E‐06
C191A,C231A	1.77E+04 ± 2.02E+03**	1.04E‐04 ± 1.54E‐05	5.86E‐09 ± 2.87E‐10*	1.83E+04	2.44E‐01	4.95E‐05
C293A,C439A	1.91E+04 ± 1.19E+03***	7.41E‐05 ± 1.23E‐05**	3.90E‐09 ± 7.80E‐10*	2.11E+04	1.91E‐01	6.45E‐05
C348A,C364A	1.40E+04 ± 5.59E+03**	1.40E‐04 ± 2.55E‐05	1.44E‐08 ± 1.36E‐08	2.38E+04	3.24E‐01	3.47E‐04
C493A,C507A	2.09E+04 ± 2.61E+03****	1.07E‐04 ± 1.12E‐05	5.11E‐09 ± 1.45E‐10*	8.65E+04	2.21E‐01	1.45E‐05
C521A,C551A	3.06E+04 ± 1.25E+03****	7.07E‐05 ± 5.60E‐06**	2.31E‐09 ± 8.74E‐11**	2.12E+05	4.52E‐01	1.42E‐05

*Note*: The kinetic constants were obtained as described in the Materials and Methods section. Mean values ±1 standard deviation represent four to eight independent experiments. The kinetic parameters and affinity of prothrombin‐argatroban binding were determined by titration of argatroban (0.1–200 μM) in a single experiment. *p < 0.05; **p < 0.01; ***p < 0.001; ****p < 0.0001 according to one‐way ANOVA analysis compared with WT. N.A., for this experiment, binding was too low to accurately assess kinetic parameters.

The kringle‐1 mutants C65A,C143A and C114A,C138A exhibited almost no binding to POmAb (Figure 5a). Because the POmAb epitope resides in kringle‐1 (Kumar et al., 2024), this lack of binding could reflect a disruption of the antibody‐binding site rather than a shift toward the closed conformation. Supporting this interpretation, addition of saturating argatroban, an active‐site ligand that promotes and stabilizes the open conformation (Pozzi, Bystranowska, et al., 2016), did not increase POmAb binding for the kringle‐1 mutants (Figure 5c). In contrast, for prothrombin wild‐type and the C191A,C231A, C293A,C439A, and C493A,C507A mutants, argatroban induced an apparent shift toward the open state as shown by an improved POmAb binding relative to measurements in the absence of argatroban. Interestingly, the C521A,C551A mutant exhibited the highest binding affinity for POmAb; however, no further enhancement was observed in the presence of argatroban. This could indicate that an unformed C521–C551 bond shifts the equilibrium toward a predominantly open conformational state.

Since our mutations may affect the argatroban affinity, we next performed prothrombin‐argatroban binding experiments to verify active‐site integrity for the ligand. Although a 10‐fold lower argatroban binding was observed for the C348A,C364A variant, surface plasmon resonance confirmed similar or better affinities for all other variants (Table 1), thereby validating our prothrombin‐POmAb binding results in the presence of argatroban.

Notably, enrichment of unformed C65–C143 and C114–C138 in the POmAb‐bound fraction (Figure 2) contrasts with our surface plasmon resonance findings, in which the corresponding mutants C65A,C143A and C114A,C138A showed minimal POmAb binding. This difference may indicate that cysteine‐to‐alanine substitution perturbs local kringle‐1 structure more severely than an unformed disulfide bond in which the cysteines are still present. Alternatively, it may indicate that specific combinations of unformed disulfide bonds give rise to conformations in which the POmAb epitope is preserved despite disruption of the C65–C143 or C114–C138 bond. Taken together, these findings provide evidence that the disulfide bond state of prothrombin, particularly the presence of unformed bonds at specific positions, is associated with adoption of the open conformation.

## 3 DISCUSSION

Growing evidence shows that proteins are produced with heterogeneous disulfide‐bonding, implying that disulfide heterogeneity is a widespread and physiologically relevant feature of mature proteins (Butera et al., 2023; Butera et al., 2025; Butera & Hogg, 2020; Pijning et al., 2021; Pijning et al., 2022). Our study demonstrates population‐level redox heterogeneity in circulating prothrombin, with all 11 assessed disulfide bonds existing in both formed and unformed states. Several bonds (C47–C60, C86–C126, C170–C248, and C219–C243) were unformed only in a minimal fraction of the protein. This suggests that these disulfide bonds represent essential elements of the prothrombin structure. Although we did not attempt to mutate these bonds, we anticipate that ablation will markedly impact secretion, as shown for factor VIII (Selvaraj et al., 2012) and von Willebrand factor (Yadegari et al., 2013). Mutation of the more unformed bonds was well tolerated, showing that cells recognize these prothrombin forms as sufficiently folded to pass quality‐control checkpoints and be secreted as mature, functional proteins. To note, structural analyses were performed on protein samples following precipitation, which, on rare occasions, may perturb the native tertiary structure and as such, may have influenced the interpretation of our findings.

Three disulfide bonds (C65–C143, C114–C138, and C191–C231) displayed a substantial discrepancy in the redox state of the two partner cysteines. A similar observation was made for the C490–C545 bond in the αIIb subunit of platelet αIIbβ3 integrin (Pijning et al., 2021). Although incomplete alkylation is a potential explanation, we observed no appreciable levels of unalkylated peptides by mass spectrometry, making this unlikely. One plausible interpretation is that “non‐native” disulfide bonds form in a subset of molecules, a phenomenon observed in IgG molecules (Liu & May, 2012), γD‐crystallin (Mora et al., 2022), and protein folding intermediates (Chang, 2011; Chang et al., 1995; Narayan, 2020). However, comprehensive identification and quantification of disulfide‐linked peptides by mass spectrometry remain technically challenging. These peptides are often detected at low abundance, and their physicochemical properties differ substantially from those of single‐cysteine peptides, limiting direct quantitative comparison with differential alkylation data. Consequently, while we cannot exclude the presence of alternative pairings, we were unable to confidently identify or quantify such species.

Another explanation relates to the high chemical reactivity of cysteines making them susceptible to a wide variety of post‐translational modifications (Chung et al., 2013). It is likely that some of the free cysteines are modified, either for specific functional purposes or to prevent undesirable disulfide formation. For instance, S‐glutathionylation of Cys33 in selenoprotein W has been reported to block formation of an intramolecular disulfide bond, altering the protein's antioxidant properties (Ko et al., 2019). Such modifications would render the cysteines unreactive to our alkylating reagent, potentially resulting in the observed discrepancy. Nevertheless, the detection of one cysteine in a pair at a substantially higher unformed fraction than its partner indicates that the corresponding disulfide bond is absent in at least that subset of circulating prothrombin molecules.

Prothrombin circulates predominantly in the closed form, which is characterized by the intramolecular collapse of kringle‐1 into the protease domain (Chinnaraj et al., 2018). This equilibrium is influenced by the active site ligand argatroban, which shifts the equilibrium significantly toward the open form (Pozzi, Bystranowska, et al., 2016) by impeding kringle‐1 interaction with the protease domain. Structural elements that impact the closed‐open conformation include the flexible linker‐2 region (Pozzi, Chen, & Di Cera, 2016) and the interaction of kringle‐1 residue Tyr93 with Trp547 in the protease domain (Chinnaraj et al., 2018). Here, we provide evidence consistent with the ablation of six of the seven prothrombin disulfides (except C348A,C364A) shifting the equilibrium toward the open conformation, indicating that these disulfide bonds contribute to stabilizing the closed state. Unformed disulfide bonds in the protease domain could induce conformational changes by altering the orientation of Trp547 and impacting its interaction with Tyr93. Similarly, unformed disulfides in the kringle domains could impact the presentation of Tyr93. It is also possible that unformed disulfides in the kringle domains result in allosteric changes within the molecule. Membrane engagement of factor Xa via its Gla domain, or by factor Va through its C domains, markedly enhances their affinity for one another, consistent with a model in which structural changes induce conformational rearrangements in distal regions of the protein (Krishnaswamy, 1990; Majumder et al., 2008; Qureshi et al., 2009; Schreuder et al., 2019). Future studies are required to elucidate these molecular mechanisms.

An important question is whether the shifts in the closed‐open equilibrium observed for our prothrombin disulfide bond variants translate into functional consequences, such as altered rates of activation by the prothrombinase complex, or changes in the pathway by which prothrombin is activated. Activation of prothrombin proceeds through two alternative pathways defined by the order of cleavage at Arg271 and Arg320 (Krishnaswamy, 2013). Initial cleavage at Arg320 generates the active intermediate meizothrombin before conversion to α‐thrombin, whereas cleavage at Arg271 initially produces the inactive intermediate prethrombin‐2 followed by proteolysis at Arg320. Assembly of prothrombinase on phospholipid membranes enforces ordered cleavage at Arg320 prior to Arg271 (Krishnaswamy et al., 1986; Orcutt & Krishnaswamy, 2004). This ordered pathway is essential for spatial control of thrombin generation. The closed conformation of prothrombin, the predominant form in circulation, was found to promote cleavage at Arg320 (Chinnaraj et al., 2018). In contrast, activation of the open form preferentially proceeds through the prethrombin‐2 pathway, indicating that the conformation of prothrombin significantly alters the presentation of the two cleavage sites toward the factor Xa active site. Considering that the majority of our disulfide bond variants adopt an open conformation, changes in their interaction with the prothrombinase complex and in the relative accessibility of the Arg271 and Arg320 cleavage sites would be expected, which may alter the canonical activation pathway. It is also important to note that the observed differences in the specific activity of our prothrombin variants as determined by aPTT do not discriminate between altered prothrombin activation, reduced thrombin activity, or impaired interaction with the prothrombinase complex as the underlying cause of the functional differences. Dedicated mechanistic studies will therefore be required to define the precise molecular basis of these effects.

An equally interesting question is whether the different covalent states of prothrombin translate to thrombin and, if so, whether these different thrombin states have functional implications. Mutation of the C521–C551 bond (C191–C220 in thrombin numbering) in thrombin was shown to perturb the sodium‐binding site and markedly decrease its proteolytic activity (Bush‐Pelc et al., 2007). Conversely, the C293–C439 bond (C1–C122 in thrombin numbering) that covalently links the A and B chains in thrombin was found to be indispensable for thrombin function, although these conclusions were drawn from reduction‐refolding experiments that may have compromised native folding (Hageman et al., 1975). Thus, structure–function relationships related to thrombin's disulfide bonds remain incompletely defined. Ongoing studies are aimed at determining the effects of the disulfide bond states on the physiological functions of prothrombin activation and thrombin activity.

Recent work identified two subpopulations of anti‐prothrombin antibodies, termed type‐I and type‐II antibodies (Chinnaraj et al., 2019). Type‐I antibodies recognize epitopes exposed when prothrombin adopts the open conformation, most notably in the kringle‐1 domain. In contrast, type‐II antibodies target regions accessible in both the open and closed conformations, such as the C‐terminal portion of the Gla‐domain (Chinnaraj et al., 2019) and the kringle‐2 domain (Pozzi et al., 2024). Structural studies using POmAb as a model type‐I antibody identified one such epitope centered around kringle‐1 residues Cys86, Leu88, Arg90‐Tyr93, and Gln110‐Glu111 (Kumar et al., 2024). This epitope overlaps or lies adjacent to the C86–C126 and C114–C138 disulfide bonds. Consistent with these mappings, our surface plasmon resonance results show that ablation of the C114–C138 bond reduces binding to POmAb. We further observed that mutation of the kringle‐1 C65–C143 disulfide bond disrupts POmAb binding. Since this disulfide is not directly located around the mapped antibody interface, this finding suggests that its ablation induces broader conformational changes within the kringle‐1 domain that indirectly impair epitope formation.

## 4 MATERIALS AND METHODS

### 4.1 Materials

All tissue culture reagents and Protein G dynabeads were from Life Technologies. The cysteine alkylating isotopes 2‐iodo‐N‐phenylacetamide (IPA) comprising a phenyl ring containing six carbon‐12 (^12^C‐IPA) or six carbon‐13 (^13^C‐IPA) were from SYNthesis med chem. All functional assays were performed in HEPES‐buffered saline (HBS: 20 mM HEPES, 0.15 M NaCl, pH 7.5) supplemented with 5 mM CaCl_2_ and 0.1% PEG8000 filtered over a 0.2 μm filter (assay buffer).

### 4.2 Blood collection and processing

Blood was obtained from healthy volunteers with the approval of The University of Sydney Human Research Ethics Committee (ethics approval 2014/244). All procedures were in accordance with the 1983 Declaration of Helsinki. Whole blood was collected into acid‐citrate‐dextrose (ACD‐A) vacutainer tubes (Becton Dickinson) and centrifuged to isolate platelet‐poor plasma. Plasma was aliquoted and stored at −80°C. Plasma aliquots were used once for experimental study.

### 4.3 Quantification of the prothrombin disulfide state

The disulfide‐bonded state of prothrombin was assessed as described (Chiu, 2019). Briefly, Protein G dynabeads (2 mg) were coated with 16 μg polyclonal anti‐prothrombin antibody (Affinity Biologicals, SAFII‐IG) in phosphate buffered saline (PBS) for 30 min at room temperature on a rotating wheel. After washing the beads three times with PBS, prothrombin samples (0.5 mL healthy donor plasma or normal pooled plasma, or 2 μg purified recombinant prothrombin in 0.5 mL 20 mM HEPES, 0.15 M NaCl, pH 7.5 supplemented with 0.1% PEG8000) were added and incubated for 1 h at room temperature. Beads were collected using a magnet and, after discarding the supernatant, were incubated with 0.3 mL of 4 mM ^12^C‐IPA in PBS containing 10% dimethyl sulfoxide (DMSO) for 1 h at room temperature in the dark. The beads were then incubated with BOLT lithium dodecyl sulfate (LDS) sample buffer (Life Technologies) containing 4 mM ^12^C‐IPA for 30 min at 60°C, and supernatants were resolved via SDS‐PAGE. For experiments without immunoprecipitation, 2 μg purified recombinant prothrombin in 30 μL 20 mM HEPES, 0.15 M NaCl, pH 7.5 supplemented with 0.1% PEG8000 or 10 μL healthy donor plasma or normal pooled plasma in 20 μL PBS were directly incubated with 4 mM ^12^C‐IPA in PBS containing 10% DMSO for 1 h for recombinant prothrombin as samples and 2 h for plasma samples at room temperature in the dark to reach complete alkylation of free cysteines. Samples were denatured with LDS for up to 60 min at 60°C before SDS‐PAGE resolution. SDS‐PAGE gels were stained with Coomassie Brilliant Blue G (Sigma‐Aldrich), and bands corresponding to prothrombin were excised, destained, dried, and treated with 40 mM DTT before washing. Gel slices were incubated with 5 mM ^13^C‐IPA for 1 h at room temperature in the dark, then washed, dried and digested overnight with 12.5 ng/μL of trypsin (Promega) in 25 mM NH_4_CO_3_ at 30°C or 12.5 ng/μL chymotrypsin (Roche) in 25 mM NH_4_CO_3_ and 10 mM CaCl_2_ at 25°C. Peptides were eluted with 5% formic acid and 50% acetonitrile, desalted using C18 Zip‐Tip (Merck Millipore), and analyzed via liquid chromatography and mass spectrometry (LC–MS). Liquid chromatography, mass spectrometry, and data analysis were performed as described (Chiu, 2019). Briefly, ~200 ng of peptides was resolved on a 35 cm × 75 μm C_18_ reverse phase analytical column using a 2%–35% acetonitrile gradient over 22 min at 250 nL/min (Ultimate 3000 HPLC; Thermo Fisher Scientific). Peptides were ionized by electrospray ionization at +2.0 kV, and tandem mass spectrometry (MS/MS) analysis was performed using a Q Exactive Plus mass spectrometer with higher energy collisional dissociation fragmentation. The data‐dependent acquisition collected MS/MS spectra of the top 10 most abundant ions with charge state ≥2 during the gradient. Data were analyzed using Mascot Daemon (Version 2.5.0, Matrix Science) against the SwissProt database with search parameters of 6 ppm precursor tolerance, ±0.6 Da product ion tolerance, and variable modifications including iodoacetanilide (Cys), ^13^C‐iodoacetanilide (Cys), and oxidation (Met). Full trypsin and chymotrypsin cleavage with up to three missed cleavages were allowed. Monoisotopic m/z values for Cys‐containing peptides (+1 to +4 charge states) were calculated using MS‐Product (v6.6.4, Protein Prospector Tools). The abundance of the redox forms of cysteines was determined from the relative ion abundance of peptides labeled with ^12^C‐IPA and/or ^13^C‐IPA. Extracted ion chromatograms were generated with XCalibur Qual Browser (v2.2.0, Thermo Fisher Scientific), and areas were calculated using the software's automated peak detection. No unlabelled (free) Cys thiols were detected in the data.

### 4.4 Construction, expression, and purification of prothrombin variants

DNA constructs encoding prothrombin (UniProt identifier P00734) variants (double cysteine to alanine substitutions of indicated disulfide bonds) were prepared from the pcDNA3.1(+) vector carrying wild‐type prothrombin by Genscript. Transfection of the plasmids encoding the prothrombin constructs into Human Embryonic Kidney (HEK)293T cells, the selection of stable clones, and the expression and purification of the prothrombin variants were performed using protocols as described previously (Schreuder et al., 2024; Toso & Camire, 2004). Briefly, prothrombin‐containing media was subjected to a Q Sepharose Fast Flow (Cytiva) column and eluted using a linear salt gradient. Prothrombin‐containing fractions were pooled and dialyzed against 20 mM Tris, 1 mM EDTA, pH 7.0 for 3 h at 4°C followed by a two‐step dialysis against 40 mM sodium phosphate, pH 6.8. Prothrombin was subsequently subjected to a Hydroxyapatite column (Bio‐Rad) and eluted using a linear gradient of sodium phosphate. Fractions containing prothrombin were pooled and precipitated by addition of 0.516 g/mL ammonium sulfate (Sigma‐Aldrich) and overnight incubation at 4°C. The following day, samples were centrifuged at 10,000*g* for 30 min at 4°C, and the supernatant was removed. Precipitate was dissolved to 5–10 mg/mL in HBS containing 0.1% PEG8000 and 50% (v/v) glycerol, and the purified prothrombin variants were stored at −30°C. Protein purity was assessed by SDS‐PAGE analysis using precast 4%–12% gradient gels under nonreducing and reducing conditions (50 mM dithiothreitol, DTT) using the MOPS buffer system (Life Technologies) followed by staining with Coomassie Brilliant Blue G (Sigma‐Aldrich).

### 4.5 Procoagulant activity of prothrombin variants

The clotting activity of purified prothrombin variants was determined using a LA‐sensitive aPTT‐based assay (Cephen LS, Stago). Normal control plasma or LA control plasma (weak and high; 50 μL), or prothrombin‐depleted plasma (45 μL) supplemented with purified prothrombin (5 μL; 100 μg/mL final) were incubated with Cephen LS reagent (50 μL), followed by a 180 s incubation period at 37°C. Coagulation was initiated with the addition of 50 μL of 25 mM CaCl_2_, upon which the coagulation time was monitored. Reference curves consisted of serial dilutions of normal control plasma that were employed to convert clotting times to mU/μg, assuming 1 U/mL prothrombin equals 100 μg/mL.

### 4.6 Open and closed conformation through chymotrypsin proteolysis

Prothrombin variants (0.1 mg/mL) were incubated at 25°C with sequencing grade chymotrypsin (Promega) at a 1:180 (w/w) ratio in 20 mM Tris, 145 mM NaCl, 10 mM CaCl_2_, pH 7.4. Samples were quenched at different time intervals with 10 μL of BOLT LDS buffer. Samples were loaded into BOLT 4%–12% Bis‐Tris protein gels run with MES buffer. Gels were stained with Coomassie Brilliant Blue G and analyzed by quantitative densitometry.

### 4.7 Surface plasmon resonance

Binding affinities of prothrombin variants for prothrombin open conformation monoclonal antibody POmAb (Kumar et al., 2024) were measured using a CM5 sensor chip in which POmAb was immobilized in flow cell 2 at ~300 RU using NHS/EDC chemistry, whereas flow cell 1 was used as a blank reference cell to correct for non‐specific binding of prothrombin variants to the inactivated CM5 sensor matrix. Dose‐dependent experiments were performed by injecting increasing concentrations (0.39–500 nM) of prothrombin variants in running buffer (20 mM HEPES, 150 mM NaCl, 5 mM CaCl_2_, 0.05% w/w Tween20, pH 7.4) at a flow rate of 30 μL/min at 25°C. Experiments evaluating POmAb binding to prothrombin variants in the presence of argatroban were performed by injecting 500 nM prothrombin variants preincubated with 200 μM argatroban. Binding affinities of prothrombin variants for argatroban were measured by immobilizing prothrombin variants on a CM5 sensor chip at ~9000 RU and injecting increasing concentrations (0.1–200 μM) of argatroban at a flow rate of 40 μL/min at 25°C. All experiments were carried out using a Biacore‐8 K+ instrument (Cytiva). After correction for baseline, association (k_on_) and dissociation (k_off_) rates were determined by fitting the sensorgrams to a 1:1 binding model, and equilibrium dissociation constants were obtained by steady‐state affinity analysis, where the binding responses were plotted as a function of prothrombin concentration and fitted to a single‐site steady‐state affinity equation in the Biacore Insight Evaluation Software (Cytiva).

### 4.8 Statistical analysis

All statistical analyses were computed using GraphPad Prism software by comparing groups using one‐way ANOVA or multiple *t*‐test analyses. Statistical significance was defined as a p‐value of <0.05 and is noted on graphs with *p < 0.05, **p < 0.01, ***p < 0.001, and ****p < 0.0001. Data are presented as individual data points, and bars represent mean values, unless stated otherwise.

## AUTHOR CONTRIBUTIONS

**Mark Schreuder:** Conceptualization; methodology; writing – original draft; writing – review and editing; investigation; formal analysis; data curation. **Diego Butera:** Methodology; investigation; data curation; formal analysis. **Suresh Kumar:** Investigation; methodology; resources. **Nicola Pozzi:** Funding acquisition; conceptualization; resources; supervision. **Philip J. Hogg:** Conceptualization; investigation; data curation; funding acquisition; writing – original draft; writing – review and editing; supervision; resources; visualization; project administration.

## Supporting information


**Table S1.** Cysteine‐containing peptides of prothrombin analyzed by HPLC and mass spectrometry. Cysteine residues are underlined in the peptide. The peptides containing the residues C17 and C22 could not be mapped via mass spectrometry. Cysteines marked in red are obtained via chymotrypsin digest, others via trypsin. Peak areas of the ^12^C‐IPA and ^13^C‐IPA‐labeled peptides ±1 standard deviation are the means of eight individual plasma samples.
**Figure S1.** Differential cysteine alkylation of the prothrombin C348 residue and peptide analysis. (A) HPLC resolution of the SPQELLCGASLISDR peptide containing C348 labeled with either ^12^C‐IPA (upper trace) or ^13^C‐IPA (lower trace). (B) Representative tandem mass spectra of the SPQELLCGASLISDR peptide. The upper and lower traces are examples of ^12^C‐IPA or ^13^C‐IPA alkylation of C348, respectively. The accurate mass spectrum of the peptide is shown in the insets (upper trace, observed [M + 2H]^2+^ = 861.43061 m/z and expected [M + 2H]^2+^ = 861.4300 m/z; lower trace, observed [M + 2H]^2+^ = 864.44025 m/z and expected [M + 2H]^2+^ = 864.4401).
**Figure S2.** Disulfide state analysis of prothrombin mutants comprising a single ablated disulfide bond. The disulfide state of eleven out of prothrombin's twelve disulfide bonds was analyzed from recombinant purified protein samples as described in ‘Materials and Methods’. The disulfide bond state of each mutant (A, C65A,C143A mutant; B, C114A,C138A mutant; C, C191A,C231A mutant; D, C293A,C439A mutant; E, C348A,C364A mutant; F, C493A,C507A mutant; G, C521A,C551A mutant) was compared with the disulfide bond state of recombinant wild‐type (WT) prothrombin. The redox state of the cysteines forming the C17‐C22 disulfide bond could not be determined. Round symbols represent the redox state of individual cysteines from a single sample, while bars indicate the mean redox state across all samples.
**Figure S3.** Electrophoretic migration of prothrombin WT and disulfide bond variants under reducing conditions. SDS‐PAGE of purified prothrombin variants (1 μg per lane) under reducing conditions visualized by staining with Coomassie Brilliant Blue R‐250. Lane 1, WT; lane 2, C65A,C143A; lane 3, C114A,C138A; lane 4, C191A,C231A; lane 5, C293A,C439A; lane 6, C348A,C364A; lane 7, C493A,C507A; lane 8, C521A,C551A. Relevant prothrombin (FII) bands and the apparent molecular weights of the standards are indicated.
**Figure S4.** SDS‐PAGE analysis of prothrombin following treatment with Peptide‐N‐Glycosidase F (PNGase). SDS‐PAGE of purified prothrombin variants (1 μg per lane) under reducing conditions visualized by staining with Coomassie Brilliant Blue R‐250. Lanes 1 & 4, WT; lanes 2 & 5, C65A,C143A; lanes 3 & 6, C114A,C138A. Lanes 1–3 depict prothrombin variants incubated for 1 h at 37°C in the absence of PNGase, lanes 4–6 depict prothrombin variants treated with 1 U of PNGase for 1 h at 37°C. Relevant prothrombin (FII) and PNGase bands and the apparent molecular weights of the standards are indicated.

## Data Availability

The mass spectrometry proteomics data have been deposited to the ProteomeXchange Consortium via the PRIDE partner repository at https://www.proteomexchange.org/. All other relevant data are available from the authors.
