# Supplementary material for: Disulfide bond status links the open and closed conformations of prothrombin
Source: Protein Sci. 2026 Nov 5;35(12):e70842. doi: 10.1002/pro.70842 (PMC13740350; doi:10.1002/pro.70842)
Supplement: Supplementary file 1 [file PRO-35-e70842-s001.docx]

**Supplementary Material**

**Disulfide bond status links the open and closed conformations of prothrombin**

Mark Schreuder, Diego Butera, Suresh Kumar, Nicola Pozzi, Philip J Hogg

**Supplementary Table 1.** Cysteine-containing peptides of prothrombin analyzed by HPLC and mass spectrometry. Cysteine residues are underlined in the peptide. The peptides containing the residues C17 and C22 could not be mapped via mass spectrometry. Cysteines marked in red are obtained via chymotrypsin digest, others via trypsin. Peak areas of the ^12^C-IPA and ^13^C-IPA-labelled peptides ± 1 standard deviation are the means of eight individual plasma samples.

| Disulfide bond | Cysteine | Peptide | ^12^C-IPA peak area | ^13^C-IPA peak area |
| --- | --- | --- | --- | --- |
| C47-C60 | C47 | YTACETAR | 3.3*10^5^ ± 1.0*10^5^ | 1.8*10^7^ ± 4.7*10^6^ |
|  | C60 | LAACLEGNCAEGLGTNYR | 2.5*10^5^ ± 6.2*10^4^ | 1.7*10^7^ ± 5.9*10^6^ |
| C65-C143 | C65 | LAACLEGNCAEGLGTNYR | 3.7*10^5^ ± 9.2*10^4^ | 1.7*10^7^ ± 5.9*10^6^ |
|  | C143 | RQECSIPVCGQDQVTVAMTPR | 2.0*10^6^ ± 6.4*10^5^ | 1.4*10^7^ ± 5.1*10^6^ |
| C86-C126 | C86 | SGIECQLWR | 2.6*10^6^ ± 9.3*10^5^ | 5.9*10^7^ ± 2.2*10^7^ |
|  | C126 | NPDSSTTGPWCYTTDPTVR | 7.2*10^5^ ± 2.4*10^5^ | 2.7*10^7^ ± 1.1*10^7^ |
| C114-C138 | C114 | CRNPDSSTTGPW | 6.3*10^6^ ± 3.0*106 | 3.9*10^8^ ± 1.1*10^8^ |
|  | C138 | RQECSIPVCGQDQVTVAMTPR | 2.1*10^6^ ± 6.5*10^5^ | 1.4*10^7^ ± 5.1*10^6^ |
| C170-C248 | C170 | SEGSSVNLSPPLEQCVPDR | 1.6*10^6^ ± 4.3*10^5^ | 5.7*10^7^ ± 1.8*10^7^ |
|  | C248 | CEEAVEEETGDGL | 6.5*10^5^ ± 3.1*10^5^ | 3.2*10^6^ ± 6.1*10^5^ |
| C191-C231 | C191 | LAVTTHGLPCLAWASAQAK | 4.4*10^6^ ± 1.2*10^6^ | 3.4*10^7^ ± 9.5*10^6^ |
|  | C231 | NPDGDEEGVWCYVAGK | 7.0*10^5^ ± 2.5*10^5^ | 3.2*10^7^ ± 1.6*10^7^ |
| C219-C243 | C219 | HQDFNSAVQLVENFCR | 8.8*10^5^ ± 2.9*10^5^ | 4.7*10^7^ ± 1.6*10^6^ |
|  | C243 | GYCDLNY | 6.1*10^4^ ± 2.3*10^4^ | 2.1*10^6^ ± 7.8*10^5^ |
| C293-C439 | C293 | TFGSGEADCGLRPLFEK | 4.2*10^6^ ± 1.2*10^6^ | 5.9*10^7^ ± 1.8*10^7^ |
|  | C439 | KPVAFSDYIHPVCLPDR | 1.6*10^6^ ± 6.4*10^5^ | 1.6*10^7^ ± 6.9*10^6^ |
| C348-C364 | C348 | SPQELLCGASLISDR | 5.0*10^6^ ± 1.3*10^6^ | 3.6*10^7^ ± 1.0*10^7^ |
|  | C364 | TAAHCL | 2.7*10^7^ ± 6.2*10^6^ | 3.8*10^8^ ± 1.5*10^8^ |
| C493-C507 | C493 | GQPSVLQVVNLPIVERPVCK | 7.6*10^6^ ± 2.0*10^6^ | 3.9*10^7^ ± 1.1*10^7^ |
|  | C507 | ITDNMFCAGYKPDEGK | 1.2*10^6^ ± 4.1*10^5^ | 6.8*10^6^ ± 2.9*10^6^ |
| C521-C551 | C521 | GDACEGDSGGPFVMK | 2.9*10^6^ ± 1.1*10^6^ | 1.5*10^7^ ± 5.8*10^6^ |
|  | C551 | WYQMGIVSWGEGCDR | 1.3*10^6^ ± 3.5*10^5^ | 6.4*10^6^ ± 2.1*10^6^ |

**Supplementary Figure 1.** Differential cysteine alkylation of the prothrombin C348 residue and peptide analysis. **(A)** HPLC resolution of the SPQELLCGASLISDR peptide containing C348 labelled with either ^12^C-IPA (upper trace) or ^13^C-IPA (lower trace). **(B)** Representative tandem mass spectra of the SPQELLCGASLISDR peptide. The upper and lower traces are examples of ^12^C-IPA or ^13^C-IPA alkylation of C348, respectively. The accurate mass spectrum of the peptide is shown in the inserts (upper trace, observed [M+2H]^2+^ = 861.43061 m/z and expected [M+2H]^2+^ = 861.4300 m/z; lower trace, observed [M+2H]^2+^ = 864.44025 m/z and expected [M+2H]^2+^ = 864.4401).

**Supplementary Figure 2.** Disulfide state analysis of prothrombin mutants comprising a single ablated disulfide bond. The disulfide state of eleven out of prothrombin’s twelve disulfide bonds were analyzed from recombinant purified protein samples as described in ‘Materials and Methods’. The disulfide bond state of each mutant (**A**, C65A,C143A mutant; **B**, C114A,C138A mutant; **C**, C191A,C231A mutant; **D**, C293A,C439A mutant; **E**, C348A,C364A mutant; **F**, C493A,C507A mutant; **G**, C521A,C551A mutant) was compared with the disulfide bond state of recombinant wild-type (WT) prothrombin. The redox state of the cysteines forming the C17-C22 disulfide bond could not be determined. Round symbols represent the redox state of individual cysteines from a single sample, while bars indicate the mean redox state across all samples.

**Supplementary Figure 3.** Electrophoretic migration of prothrombin WT and disulfide bond variants under reducing conditions. SDS-PAGE of purified prothrombin variants (1 μg per lane) under reducing conditions visualized by staining with Coomassie Brilliant Blue R-250. Lane 1, WT; lane 2, C65A,C143A; lane 3, C114A,C138A; lane 4, C191A,C231A; lane 5, C293A,C439A; lane 6, C348A,C364A; lane 7, C493A,C507A; lane 8, C521A,C551A. Relevant prothrombin (FII) bands and the apparent molecular weights of the standards are indicated.

**Supplementary Figure 4.** SDS-PAGE analysis of prothrombin following treatment with Peptide-N-Glycosidase F (PNGase). SDS-PAGE of purified prothrombin variants (1 μg per lane) under reducing conditions visualized by staining with Coomassie Brilliant Blue R-250. Lanes 1 & 4, WT; lanes 2 & 5, C65A,C143A; lanes 3 & 6, C114A,C138A. Lanes 1-3 depict prothrombin variants incubated for 1 hour at 37 °C in the absence of PNGase, lanes 4-6 depict prothrombin variants treated with 1U of PNGase for 1 hour at 37 °C. Relevant prothrombin (FII) and PNGase bands and the apparent molecular weights of the standards are indicated.
